# The Relationships between Cholesterol and Suicide: An Update

**DOI:** 10.5402/2012/387901

**Published:** 2012-12-23

**Authors:** Domenico De Berardis, Stefano Marini, Monica Piersanti, Marilde Cavuto, Giampaolo Perna, Alessandro Valchera, Monica Mazza, Michele Fornaro, Felice Iasevoli, Giovanni Martinotti, Massimo Di Giannantonio

**Affiliations:** ^1^Psychiatric Service of Diagnosis and Treatment, Department of Mental Health, “G. Mazzini” Hospital, NHS, ASL 4, 64100 Teramo, Italy; ^2^Department of Neurosciences and Imaging, “G. D'Annunzio” University, 66013 Chieti, Italy; ^3^Pharmaceutical Service, “G. Mazzini” Hospital, NHS, ASL 4, 64100 Teramo, Italy; ^4^IASM, 67100 L'Aquila, Italy; ^5^Department of Clinical Neurosciences, Villa San Benedetto Hospital, Hermanas Hospitalarias, 22032 Albese con Cassano, Italy; ^6^Villa S. Giuseppe Hospital, Hermanas Hospitalarias, 63100 Ascoli Piceno, Italy; ^7^Department of Health Science, University of L'Aquila, 67100 L'Aquila, Italy; ^8^Department of Formative Sciences, University of Catania, 95100 Catania, Italy; ^9^Laboratory of Molecular Psychiatry and Psychopharmacotherapeutics, Section of Psychiatry, Department of Neuroscience, School of Medicine, University of Naples “Federico II”, 80121 Naples, Italy

## Abstract

Cholesterol is a core component of the central nervous system, essential for the cell membrane stability and the correct functioning of neurotransmission. It has been observed that cholesterol may be somewhat associated with suicidal behaviours. Therefore, the aim of this paper was to elucidate current facts and views about the role of cholesterol levels in mood disorders. The majority of the studies reviewed in the present paper suggest an interesting relationship between cholesterol (especially lower levels) and suicidality. On the other hand, particularly during the last years, relationships between serum cholesterol and suicidality were doubted on the basis of some recent studies that have not found any correlation. However, the debate on relationships between cholesterol and suicide is open and longitudinal studies on a larger sample of patients are needed to further clarify this important issue.

## 1. Introduction

Cholesterol is a core component of the central nervous system (CNS), essential for the cell membrane stability and the correct functioning of neurotransmission [1]. It plays a crucial role in the second messenger systems of the brain that have been associated with the mechanism of action of antidepressant drugs and mood stabilizers and may be involved in the aetiology and pathogenesis of mood disorders [2, 3]. Several studies have shown that patients with major depression (MD) have lower total cholesterol (TC) levels than nondepressed individuals [4, 5]. In addition, lower high-density lipoprotein (HDL-C) cholesterol concentrations and higher ratios of TC/high-density lipoprotein (TC/HDL-C) and low-density lipoprotein/high-density lipoprotein (LDL-C/HDL-C) are also noted in patients with MD [6–8]. It has been observed that cholesterol may be somewhat associated with suicidal behaviours [9, 10]. Moreover, it was found that adiposity together with a high waist-to-hip ratio may be associated with an increased risk of suicide [11].

All considered, a possible usefulness in evaluating serum cholesterol levels in affective patients may be the prediction of development of suicidal ideation [12]. Therefore, the monitoring of cholesterol may result in a reduction of mortality due to suicidality [9]. However, during the last two years, involvement of serum cholesterol in pathogenesis of MD was doubted on the basis of some recent studies that have not found any correlation between serum cholesterol and depressive symptoms [13].

Therefore, the aim of the present paper was to elucidate current facts and views about the role of cholesterol levels in suicidality.

## 2. Studies That Found an Association between Cholesterol and Suicidality

Despite the controversial results about relationships between cholesterol levels and depressed mood, the majority of the literature on this subject seems to show a consistent relationship between cholesterol and suicide [14–20]. The possible relationships between cholesterol levels and suicidality were initially suggested when an excess mortality for violence (suicides and injuries) was observed following the use of cholesterol-lowering drugs [21, 22]. However, even if a meta-analysis conducted by Muldoon et al. [23] showed that deaths from suicides, accident, and violence were not significantly increased among participants randomised to a cholesterol-lowering intervention compared with those in the control groups, researchers' attention was focused on this topic.

Despite some lines of evidence of a link between higher cholesterol levels and increased suicidality [11, 24], mainly, as well as for MD, a relationship was found between lower serum cholesterol levels and suicidality [25–27] especially in male gender [28]. Gallerani et al. [29] published data from a controlled population of 331 parasuicides and found a lower cholesterol level in this group. Sullivan et al. [27] investigated the association between TC and suicidality in a sample of 90 men and women with MD and found a significant association between lower cholesterol levels and increased suicidality. These findings were confirmed by Kunugi et al. [30] who similarly found a relationship between low serum cholesterol and suicide attempts and by Papassotiropoulos et al. [31] who reported that the risk of acute suicidality decreased with increasing TC levels irrespective of age, gender, and nutritional status. Rabe-Jabłońka and Poprawska [32] determined the level of TC and LDL-cholesterol in blood samples taken from 102 patients with recurrent major depression (according to Diagnostic and Static Manual of Mental Disorders, 4th Edition, DSM-IV) and performed analyses during the acute period of major depression in 3 subgroups: with and without suicidal ideation (S+, S−), and after suicidal attempts (AS), and during remission of depressive symptoms. Moreover, it has been demonstrated that, in acutely depressed patients, low TC and LDL-cholesterol were significantly associated with higher suicide risk. Recently, Olié et al. [33] tested the clinical applicability of a potential cut-off value in the evaluation of suicidal risk. They found that the risk of suicide attempts increased 7.33-fold in men and 15.6-fold in women in the lowest cholesterol quartile compared to subjects in the highest quartile. Thus, serum cholesterol level may be a strong risk factor for suicidal behavior in patients with depressive symptoms. The authors suggested that total serum cholesterol levels measured at admission may be a useful biological marker of suicidal risk. Also Ruljancic et al. [34] reported lower serum cholesterol in a sample of MD patients with attempted suicide compared to MD patients without attempted suicides. In a recent study, Troisi [35] found that attentional impulsiveness (i.e., the inability to focus attention or concentrate, related to executive function deficits and reduced cognitive flexibility), a demonstrated risk factor for suicide, was associated with lower cholesterol levels.

Interestingly, there are some studies that found a low serum cholesterol level especially in violent suicide attempts [36–38]. Vevera et al. [39] found that patients with a violent suicidal attempt had significantly lower cholesterol levels than patients with nonviolent attempts and the control subjects. They suggested that low levels of cholesterol may be associated with increased tendency for impulsive behaviour and aggression and contribute to a more violent pattern of suicidal behaviour. Tripodianakis et al. [40] provided further confirmation to this hypothesis and found lower serum cholesterol in violent suicide attempters together with an increased noradrenaline turnover in such subjects. Further observations about relationships between impulsivity and violent behaviour seem to bring other indirect evidence to the connection between lower cholesterol levels and violent suicide attempts [41, 42].

The observation of lower serum cholesterol levels associated with violent suicide attempts seems also to pertain to schizophrenia. In fact, Marčinko et al. [43] found that schizophrenic patients with a violent suicidal attempt showed significantly lower cholesterol levels and significantly higher cortisol level than patients with nonviolent attempts and control subjects. These findings can be indicative of a more generalized involvement of lower cholesterol levels in violent suicide also in other psychiatric disorders than MD. In fact, concerning suicidality, similar results were found in anorexia nervosa [44], panic disorder [45], dissociative disorder [46], and borderline personality disorder [47].

It has also been demonstrated that lower cholesterol levels were associated with lifetime suicide attempts [48]. This observation was confirmed by the finding of another study that showed a moderate positive association between cholesterol, triglycerides, body mass index (BMI), and suicide attempts in subjects with depressive symptoms during the past 12 months [49]

Moreover, it has been reported that lower HDL-C levels (≤40 mg/dL) were associated with an increased prevalence of suicide attempts in women [50]. The finding of a lower HDL-C level in suicide attempters was also confirmed in other studies [51, 52]

Recently, it has been suggested that alexithymia (a personality style characterized by difficulty in identifying and describing feelings together with concrete and poor introspective thinking, frequently but not necessarily associated with depressive symptoms) may be related with cholesterol dysregulation and increased suicidality. In particular, alexithymic individuals with MD and higher suicide risk may show lower HDL-C [53] and higher triglyceride levels [54].

## 3. Neurobiological Mechanism of the Relationships between Cholesterol and Suicidality

In 1992 Engelberg [55] presented a hypothesis linking cholesterol and the serotonergic system. He hypothesized that a reduced serum cholesterol level may be accompanied by changes in viscosity and function of serotonin receptors and transporters as well as by decreased serotonin precursors that may cause an increase in suicide ideation. Maes et al. [56] focused their attention on the phenotype of haptoglobin connected with chromosome 16, where the gene encoding lecithin-cholesterol acetyltransferase (LCAT)—the enzyme catalyzing cholesterol esterification in the blood—is located. The authors hypothesized that decreased concentration of esterified cholesterol might be connected with the defect of chromosome 16 that may increase susceptibility to depression by changes in the viscosity of the cellular membrane increasing the risk of aggressive and autoaggressive behaviour. 

As consequence of decreased lipid microviscosity in neural membranes, serotonin receptor exposure on the membrane surface may be reduced, with an hypofunction of such receptors. The serotonergic system is strongly recognized as being linked to suicidality and impulsive and aggressive behaviour as lower concentrations of 5-hydroxyindolacetic acid (5-HIAA) in the cerebrospinal fluid (CSF) in suicides and suicide attempters were found in several studies [57]. Jokinen et al. [52] found a significant positive correlation between serum total cholesterol and level of CSF 5-HIAA in suicide attempters that remained significant after correction for age, gender, BMI, and comorbid substance abuse, even if earlier studies did not find an association [58]. These findings were more recently confirmed by Hibbeln et al. [59] who evaluated TC and CSF 5-HIAA in 42 drug-naïve suicide attempters (with blood samples acquired and lumbar punctures performed in a standardized manner in a controlled study setting) and found a significant positive correlation between serum total cholesterol and level of CSF 5-HIAA. 

As suggested by Jokinen et al. [52], it is also possible that the relationship between cholesterol and the serotonergic system may be a two-way relationship, hypothesis corroborated by the study of Fischer et al. [60] who found an association between higher LDL levels and the long allele coding for serotonin transporter gene in women.

## 4. Studies That Did Not Find an Association between Cholesterol and Suicidality

However, especially during the last years, involvement of serum cholesterol in pathogenesis of suicide was doubted on the basis of some studies [61, 62]. For instance, results of a retrospective study conducted on 213 psychiatric inpatients (including 61 patients with affective disorders) did not find significant differences in the serum cholesterol levels between patients who had and had not made a suicide attempt after controlling for age or body mass index [63]. In a prospective study on 92 inpatients with an MD episode at admission, regardless of a significant reduction in depression and suicidality scores, analyses of serum lipid concentrations after 1 and 4 weeks of antidepressant treatment showed no significant differences in lipid levels between patients with and without a history of attempted suicide, even if in patients who used a violent suicide method, there was a trend for lower TC levels compared to those with nonviolent attempt [64]. Further evidence on the lack-of-association hypothesis was also provided by Huang [65] who evaluated serum lipid profiles, during a 2-year period, in 168 subjects (109 patients with MD and 59 healthy controls) and found that, even if serum lipid profile changes in patients with MD during acute phase were observed, there were no significant differences of any kind in serum lipid profiles between MD patients with or without suicide attempts. The lack of association was also confirmed in a sample of suicidal borderline personality disorder patients who showed no serum cholesterol levels differences between nonsuicidal patients and healthy controls [66]. Moreover, Pompili et al. [67] reported that nearly lethal resuscitated suicide attempters had no low serum levels of cholesterol and triglycerides compared to patients who had not made a recent suicide attempt.

More recently, de Leon et al. [68] evaluated 193 current suicide attempters and found that low cholesterol levels were not associated with increased suicide risk but with a decreased risk in men. The lack-of-association hypothesis was substantially confirmed also by Persons et al. [69].

More recently, D'Ambrosio et al. [70] did not find any association between serum lipid levels and suicide in patients with bipolar disorder.

## 5. Conclusions

The majority of the studies reviewed in the present paper suggested an interesting relationship between cholesterol levels and suicidality, even if a further elucidation of a possible pathophysiological mechanism with regard to cholesterol and suicidality is necessary in future studies. On the other hand, especially during the last years, relationships between serum cholesterol and suicidality were doubted on the basis of some recent studies that have not found any correlation. Although, to date, it is hard to draw a clear conclusion on this topic, it is possible to argue, on the basis of precision of measurements and experimental designs of reviewed studies, that the accuracy of earlier studies, that is, those supporting the hypothesis of a link between cholesterol levels and suicide, was somewhat lower than more recent studies that did not find any relationship. Moreover, it has been observed that changes in plasma cholesterol may be shared by other clinical entities, not clearly corresponding to a peculiarity of suicidality. However, the debate on relationships between cholesterol and suicide is open and longitudinal studies on a larger sample of patients are needed to further clarify this important issue, even if the time to get any conclusive data using longitudinal designs in items such as suicide (among many others) may take longer than the lifespan of the researcher.

## Abbreviations

TC: Total cholesterol
HDL-C: High-density lipoprotein cholesterol
CNS: Central nervous system
MD: Major depression
LDL-C: Low-density lipoprotein cholesterol
DSM-IV: Diagnostic and Static Manual of Mental Disorders, 4th Edition
BMI: Body mass index
LCAT: Lecithin-cholesterol acetyltransferase
5-HIAA: 5-Hydroxyindolacetic acid
CSF: Cerebrospinal fluid.
